# Supported Decision Making in the Prevention of Compulsory Interventions in Mental Health Care

**DOI:** 10.3389/fpsyt.2019.00137

**Published:** 2019-03-29

**Authors:** Martin Zinkler

**Affiliations:** Kliniken Landkreis Heidenheim gGmbH, Department of Psychiatry, Psychotherapy and Psychosomatic Medicine, Teaching Hospital of Ulm University, Heidenheim, Germany

**Keywords:** supported decision-making, Compulsory detention, Coercive treatment, Psychiatry, Human Rights

Several strands of thought, international law and clinical practice shaped the emergence of supported decision making in mental health care: the UN Convention on the Rights of Persons with Disabilities, in particular Article 12 on Equal Recognition before the Law (1), the General Comment No.1 of the UN Committee on the Rights of Persons with Disabilities (2) emphasizing “support in the exercise of legal capacity” and obliging states “to replace regimes of substitute decision-making by supported decision-making, which respects the person's autonomy, will and preferences,” the introduction of shared decision making in medicine (3), and the users' movement challenging traditional paternalistic approaches in psychiatry (4).

Within psychiatry the uptake of the convention with supported decision making was rather hesitant and perceived as challenging (5). Some commentators went as far as suggesting “an urgent consideration (of the General Comment No.1) with the full participation of practitioners” (6). Rather than extending this discussion, I will look at how supported decision making could work in the treatment of severe depression and psychosis, with the aim to prevent coercive interventions.

Arguably, the widespread use of detention, coercion and isolation is a major obstacle for users of mental health services to perceive their service as trustworthy and helpful. Particularly coercive medication may have negative consequences on subsequent service use, as it is strongly linked to disapproval of treatment (7) or associated with lower acceptance of any form of containment (8). With an emphasis on will and preferences, supported decision making should have some potential in reducing coercive interventions in mental health settings.

## Severe Depression

Few clinicians will experience difficulties with supported decision making in the treatment of mild or moderate depression. However, acute mental or general health services will also encounter patients who want to end their lives or perceive themselves as unworthy of any treatment and would therefore prefer to be discharged home and left to themselves. A traditional approach would be to ask these patients to remain in hospital until they feel better. If a capacity assessment takes place, an “impaired decision making capacity” (5) may be found. Hence, a “doctor-knows-best” approach or a functional approach to capacity will provide an ethical or legal justification to keep the patient in hospital.

On the contrary, looking at will and preferences the clinician may first encounter the actual will of the person: “I don't want to remain in hospital.” Before that however, the preferences of the person were to remain alive and well, otherwise she may have ended her life or suffered severe harm from illness before the current situation emerged. Actual will and hitherto expressed preferences seem to point in different directions.

Clearly, according to the CRPD the person is entitled to support. Support may involve information on the possible outcomes of depression (mostly positive) and treatment options (usually available) based on will *and* preferences. A patient may agree to treatment at home with a crisis resolution team as an alternative to hospital admission (9). This option may be available in some places, in others a move toward supported decision making may drive service development toward person-centered care.

But what if the person simply wants to end it all? Will A&E services just leave her alone and provide some information on counseling and outpatient treatment options? How can will *and* preferences (10) determine treatment and support? The treatment team will look at the current will of the person and previous expressions of will and preferences. They will look for advance directives or joint crisis plans (11), for a power of attorney and informal support arrangements in the family. Friends and family can provide information on previously expressed preferences.

This may take some time. Therefore, until a thorough investigation of the person's will and preferences has taken place, the person should be kept safe, even if this goes against the will expressed in that particular time and situation. Any action against the current will of the person should be scrutinized by a court of law to make sure it is proportional and represents the least restrictive option. The court would not look at an assessment of capacity but establish a contradiction between will and preferences and point out a way forward to resolve this. Keeping the patient safe is not a legitimate reason for coercive treatment; as far as medical treatment is concerned, the will of the person (not to be medically treated) will be respected.

The court may well suggest that the hospital offers treatment at home or day hospital treatment as an alternative to inpatient treatment (and a less restrictive option) if the person does not want to remain in hospital. For a patient in hospital the court may suggest that the hospital offers 1:1 support to allow the patient to leave the hospital for walks, for physical exercise or to buy some items (in order to minimize the infringement on the person's rights).

The court may order a detention in hospital for just a week or two, before will and preferences are reviewed. Will and preferences may go against treatment of depression, therefore specific treatment cannot be given. On the other hand, the court may suggest that the hospital finds out more about will and preferences by engaging the patient in individual sessions of supported decision making: clinicians and peer support workers (staff with first-hand experience of depression) (12) share their experience (in the role of someone treating depression or someone suffering from depression), aim at an understanding of the patient's preferences and inform the patient on therapeutic options. Family and friends can be counseled to support the patient.

Traditionally, hospital treatment may start with an explanation: “our assessment shows that you are suffering from a severe depressive episode. The treatment options are psychotherapy and antidepressant medication. As long as you harbor suicidal thoughts, the treatment should be as an inpatient before we look at other treatment options as an outpatient or in a day hospital.” However, respecting will and preferences leads to a different approach: “we are here to support you at this critical moment in your life. While you would rather want to be discharged home and left alone, we wonder whether this really is the time, considering your life as a whole and the current situation where you want to end it all. We would therefore want to support you for a little while, perhaps a week or two, until we are clear about your will and about your preferences for your life. Be assured, no treatment will be given against your will.”

## Psychosis

Around 60% of patients involuntarily admitted to hospital are diagnosed with psychosis (13). Shifting from *detention and coercion in hospital* toward *supported decision making* would, on one hand, mark a massive change for people diagnosed with psychosis in their treatment experience. On the other hand, it would confront hospital staff with situations where detention or coercion are no longer viable or are far more restricted than now.

Across different legislations people diagnosed with psychosis are admitted involuntarily on the basis of imminent harm to themselves, imminent harm to others, a medically determined need for treatment, or a (medically determined) lack of capacity to consent to treatment, or a combination of these criteria (14). As mentioned before, applying these criteria to justify detention in hospital or involuntary treatment seems to be in contrast with General Comment No.1 on Article 12 of the CRPD (2).

Similarly, using the example of depression, supported decision making would start with an assessment of will and preferences. Where no will is expressed and the preferences are not known, treatment may begin on the basis of “best interpretation of will and preferences” (2). More commonly, the actual will may point to discharge from hospital. In this case, it needs to be established if the request to be discharged from hospital represents a will not to be supported at all, or if support would be accepted in a less restrictive or less institutional context: as an outpatient, at home, in a day hospital, on a medical rather than a psychiatric ward. Once the setting of support is decided, the content of treatment can be negotiated.

But what should be done if imminent harm to the person or to another person is at stake? There may be a court order requiring the person to remain in hospital for assessment. The clinical team would use this time to establish will and preferences and inform the court if these go against treatment in hospital. The court would then have to decide if a further stay in hospital is warranted (in order to avoid imminent harm, or to allow more time to establish will and preferences). However, will and preferences going against hospital treatment would eventually lead to discharge from hospital.

Yet the obligation to support the person does not stop with discharge from hospital. The need for support may still be high and services like intensive case management (ICM) or assertive community treatment (ACT) may have to be put in place (15). However, compulsory community treatment, which in the UK or the US often combines with ICM or ACT (16) would not be consistent with the principle of Article 12 of the CRPD.

To support people with psychosis, clinicians need effective means of communication. Often, at least at the beginning of treatment and based on previous experiences with mental health services, patients find it hard to trust their doctors and nurses. Building trust between a treatment team and a person with psychotic symptoms needs time and patience. Coercive interventions on the other hand are likely to damage that trust.

To build therapeutic relationships, mental health services need to provide a safe environment, time, and therapeutic expertise. They should strive to avoid any coercive interventions and should involve family and friends of the person concerned. The World Health Organization recommends the “Open Dialogue” approach as a specific alternative to traditional mental health services “to support the individual's network of family and friends, as well as (to) respect the decision-making of the individual”(17). “Open dialogue” is a flexible service for the treatment of psychosis in a community context not only with the potential to avoid coercive interventions and hospital admissions but also to improve the outcomes of psychosis (18).

For people experienced with mental health services, the options to draft an advance statement or to agree on a joint crisis plan with their respective mental health services will help to avoid uncertainty about their will and preferences in situations when communication becomes difficult (10). In exceptional cases of psychosis, weeks or even months may pass with uncertainty on will and preferences. A court may have to decide on the proportionality of curtailing civil liberties against other considerations at stake.

## Conclusion

Based on the General Comment on Article 12 of the CRPD (2), supported decision making may hold potential in replacing substitute decision making and in reducing coercive interventions in mental health care. To implement supported decision making in clinical practice, it should not stop at capacity assessments or at situations where the health and safety of the person concerned are at risk. Promising approaches in the support of people with severe mental illness are the Open Dialogue model (18), Advance Statements (11) and Crisis Resolution/Home Treatment Teams (9). Based on their lived experience with mental health problems, peer support workers are in a unique position to support professionals in eliciting will and preferences to guide treatment and support. Clinical techniques in building trustful relationships and in effective communication with people suffering from psychosis and depression need improving. Mental health care research and clinical services should embrace this challenge.

## Author Contributions

The author confirms being the sole contributor of this work and has approved it for publication.

### Conflict of Interest Statement

The author declares that the research was conducted in the absence of any commercial or financial relationships that could be construed as a potential conflict of interest.
